# Dexamethasone and BCAA Failed to Modulate Muscle Mass and mTOR Signaling in GH-Deficient Rats

**DOI:** 10.1371/journal.pone.0128805

**Published:** 2015-06-18

**Authors:** Hikaru Nishida, Ayaka Ikegami, Chiaki Kaneko, Hitomi Kakuma, Hisano Nishi, Noriko Tanaka, Michiko Aoyama, Makoto Usami, Yasuhiko Okimura

**Affiliations:** 1 Department of Nutrition and Food Science, Kobe Women’s University Graduate School of Life Sciences, Kobe, Japan; 2 Division of Nutrition and Metabolism, Kobe University Graduate School of Health Sciences, Kobe, Japan; University of Rome La Sapienza, ITALY

## Abstract

Branched-chain amino acids (BCAAs) and IGF-I, the secretion of which is stimulated by growth hormone (GH), prevent muscle atrophy. mTOR plays a pivotal role in the protective actions of BCAA and IGF-1. The pathway by which BCAA activates mTOR is different from that of IGF-1, which suggests that BCAA and GH work independently. We tried to examine whether BCAA exerts a protective effect against dexamethasone (Dex)-induced muscle atrophy independently of GH using GH-deficient spontaneous dwarf rats (SDRs). Unexpectedly, Dex did not induce muscle atrophy assessed by the measurement of cross-sectional area (CSA) of the muscle fibers and did not increase atrogin-1, MuRF1 and REDD1 expressions, which are activated during protein degradation. Glucocorticoid (GR) mRNA levels were higher in SDRs compared to GH-treated SDRs, indicating that the low expression of GR is not the reason of the defect of Dex’s action in SDRs. BCAA did not stimulate the phosphorylation of p70S6K or 4E-BP1, which stimulate protein synthesis. BCAA did not decrease the mRNA level of atrogin-1 or MuRF1. These findings suggested that Dex failed to modulate muscle mass and that BCAA was unable to activate mTOR in SDRs because these phosphorylations of p70S6K and 4E-BP1 and the reductions of these mRNAs are regulated by mTOR. In contrast, after GH supplementation, these responses to Dex were normalized and muscle fiber CSA was decreased by Dex. BCAA prevented the Dex-induced decrease in CSA. BCAA increased the phosphorylation of p70S6K and decreased the Dex-induced elevations of atrogin-1 and Bnip3 mRNAs. However, the amount of mTORC1 components including mTOR was not decreased in the SDRs compared to the normal rats. These findings suggest that GH increases mTORC1 activity but not its content to recover the action of BCAA in SDRs and that GH is required for actions of Dex and BCAA in muscles.

## Introduction

A variety of diseases and conditions, including sepsis, cancer, renal failure, excessive glucocorticoids and denervation and disuse of the muscle, result in muscle atrophy. Muscle atrophy decreases mobility, increases susceptibility to injuries and reduces the quality of life [1]. Additionally, muscle mass loss leads to altered glucose and lipid metabolism and decreased energy expenditure [2, 3]. Therefore, protecting against muscle atrophy is important for maintaining favorable conditions for life.

Skeletal muscle mass is determined by the balance between the synthesis and degradation of muscle proteins. Several hormones and nutrients, such as branched-chain amino acids (BCAAs), stimulate protein synthesis via the activation of the mammalian target of rapamycin (mTOR). mTOR forms the mTOR complex 1 (mTORC1) with Raptor, GβL, PRAS40 and DEPTOR and phosphorylates 4E-binding protein 1 (4E-BP1) and p70 S6 kinase (p70S6K). Phosphorylated 4E-BP1 and p70S6K stimulate protein synthesis [4]. mTORC1 plays roles in the prevention of protein degradation in addition to protein synthesis. The ubiquitin-proteasome and autophagy systems are two major degradation pathways of cellular proteins and are activated in muscle atrophy [5]. The majority of types of muscle atrophy, including glucocorticoid-induced muscle atrophy, are related to increases in the expressions of atrogin-1 and MuRF1, which are muscle specific ubiquitin ligases. Atrogin-1 and MuRF1 stimulate the ubiquitination of target proteins that are then degraded in proteasomes, which results in the development of muscle atrophy [6]. Glucocorticoids also increase Bnip3, which is a pro-apoptotic protein that can induce autophagy and stimulates protein degradation. mTORC1 suppresses the expressions of atrogin-1, MuRF1 and Bnip3 [7]. In contrast, glucocorticoids up-regulate REDD1, which inhibits mTORC1 activity [8, 9]. Hence, glucocorticoids partially stimulate muscle atrophy via the inhibition of mTORC1 activity. In addition to REDD1, Foxo1 and MuRF1 are direct targets of glucocorticoid and involved in muscle atrophy [10–12].

In contrast to the glucocorticoid actions that stimulate muscle atrophy, growth hormone (GH), thyroid hormone and testosterone exert protective actions against muscle atrophy [13–15]. GH is secreted from the pituitary gland, binds to cell membrane receptors in target organs, and regulates cell growth, differentiation and metabolism in various organs [13, 16]. Skeletal muscle is one of the target organs of GH, and muscle atrophy and related metabolic disorders are induced by attenuations of GH secretion [17].

GH stimulates insulin-like growth factor 1 (IGF-1) secretion in a variety of organs. The liver is the primary organ that produces IGF-1 and produces 70% of the circulating IGF-1. In contrast, GH increases IGF-1 production in muscles [18]. It is thought that locally produced IGF-1 is more important than GH or circulating IGF-1 for muscle development and protection against muscle atrophy based on analyses of several mouse models with reduced GH and/or IGF-1 signaling [19–21]. IGF-1 activates the phosphoinositide 3-kinase (PI3K) pathway. This pathway stimulates protein synthesis and cellular proliferation [22]. When the PI3K pathway is activated, mTOR is phosphorylated and activates mTORC1 [23], leading to the stimulation of protein synthesis and the attenuation of protein degradation.

BCAAs (i.e., leucine, isoleucine, and valine) also exert a protective effect against muscle atrophy. We have previously reported that orally administered BCAA increases the muscle weight and cross-sectional area (CSA) of the muscle in rats [24]. Additionally, we have reported that the oral administration of BCAA inhibits dexamethasone (Dex)-induced muscle atrophy in rats [24], and Shimizu et al. reported that BCAA suppresses muscle atrophy [7]. We have also reported the effectiveness of BCAA in protecting against muscle atrophy in a hindlimb suspension-induced muscle atrophy rat model [25]. mTOR plays an important role in these actions of BCAA. mTOR is involved in the protein synthesis that is stimulated by BCAA and in the protein degradation that is suppressed by BCAA [26, 27]. Several mechanisms by which BCAA stimulates mTOR activity have been proposed, although the precise mechanism remains unclear. The proposed pathway by which mTOR action is stimulated is not the same as the pathway utilized by IGF-1 and is not dependent on PI3K [28]. These findings suggest a possible protective action of BCAA against muscle atrophy in the absence of GH/IGF-1. We hypothesized that BCAA would exert a protective effect against Dex-induced muscle atrophy in GH-deficient rats and tested this hypothesis in the present study. However, unexpectedly, we found that Dex and BCAA failed to modulate muscle mass and mTOR signaling in GH-deficient rats and that GH reversed the actions of Dex and BCAA.

## Materials and Methods

### Animals

Sprague—Dawley (SD) rats (6-week-old, weighting 180–200 g) and spontaneous dwarf rats (SDRs; 6-week-old, weighting 45–55 g) were purchased from Japan SLC (Hamamatsu, Japan). SDRs are GH-deficient rats due to a splicing abnormality of the GH gene, and plasma GH is undetectable in these rats. The animals were housed in a room at 22°C on a 12-h light-dark cycle, provided food and water ad libitum, and used for experiments after one week of preliminary rearing. This study was approved by the Committee on Animal Experimentation of Kobe Women’s University (Permit Number: 13A6)and performed in accordance with the guidelines of the animal ethics committee of Kobe Women’s University. All efforts were made to minimize the number of animals used and their suffering.

### *In vivo* study

#### Experiment 1

To examine the effects of Dex and BCAA on muscle in the SDRs, BCAA (600 mg/ kg body weight/ day, LIVACT, a brand name of BCAA granules that consist of 46% leucine, 28% valine, and 23% isoleucine, Ajinomoto, Tokyo, Japan), Dex (600 μg/ kg, Nacalai Tesque, Kyoto, Japan) or the combination of both was administered to 7-week-old SDRs for 5 days as described previously [24]. Briefly, 20 SDRs were divided into four groups of five animals each. The first group received intraperitoneal injections of Dex once per day at 18:00. The second group received Dex and an additional administration of BCAA. The third group received only BCAA. In the fourth control group, Dex and BCAA were replaced by equivalent volumes of saline (0.9% NaCl) and water, respectively. The dose of Dex was determined based on previous studies [24, 29]. BCAA was dissolved in water. The rats were allowed free access to the fixed amount of BCAA solution (600 mg/ kg) from 18:00 to 9:00. All the BCAA solution had been drunk by 9:00, water was then supplied to the rats. The body masses of the rats were measured every day at 13:00. After 5 days of treatment with Dex, BCAA or both, the soleus and extensor digitorum longus (EDL) muscles were collected at 12:00 under anesthesia (medetomidine 0.3 mg/ kg, midazolam 4.0 mg/ kg and butorphanol 5.0 mg/ kg) for analysis. The SDRs, then, were euthanized by intraperitoneal injection of pentobarbital (150 mg/ kg).

#### Experiment 2

To clarify whether GH supplementation affected the actions of BCAA and Dex on the SDR muscles, GH was continuously administered. Twenty-four 6-week-old SDRs were anesthetized, and osmotic minipumps (model 2002, Alzet, CA, USA) were implanted subcutaneously. These pumps contained porcine GH (pGH, 10 μg/ μl, NIH National Hormone & Peptide Program). pGH was administered at a rate of 5μg / h for 14 days (days 1–14). This dose of GH was selected to restore the GH and IGF-1 levels to within the physiological ranges [30]. BCAA, Dex or the combination of the two was administered according to the same protocol described in Experiment I for 5 days (days 10–14), and the soleus, EDL and rectus femoris muscles were collected under anesthesia with medetomidine, midazolam and butorphanol on day 15. The SDRs, then, were euthanized by intraperitoneal injection of pentobarbital (150 mg/ kg).

#### Experiment 3

To compare the quantities of mTORC1 subunits and the molecules upstream and downstream of mTORC1 between the SDR and SD rats, the soleus and rectus femoris muscles were collected from six 6-week-old SDR and five 6-week-old SD rats under anesthesia with medetomidine, midazolam and butorphanol. The SDR and SD rats, then, were euthanized by intraperitoneal injection of pentobarbital (150 mg/ kg).

### Histological analysis

The soleus and EDL muscles were embedded in tragacanth gum (Nacalai Tesque), frozen in acetone chilled with dry ice and sliced at a thickness of 10 μm using a cryostat. The resulting transverse sections were examined by ATPase staining (pH 10.7). The CSAs of 500 muscle fibers per muscle from each rat were analyzed using Scion Image software.

### Protein extraction and western blotting

Muscle homogenates were prepared in a 1:10 ratio of ice-cold homogenizing buffer (20 mM Tris pH 8.0, 1% Nonidet-P-40, 120 mM NaCl, 20 mM NaF, 1 mM ethylene diamine tetraacetic acid (EDTA), 1 mM ethylene glycol tetraacetic acid (EGTA), 30 mM β-glycerophosphate, 15 mM sodium dihydrogen pyrophosphate, 2 mM sodium orthovanadate and Protease Inhibitory Cocktail (Nacalai Tesque, Kyoto, Japan)) using a polytron homogenizer. After centrifugation at 13,000 g for 25 min at 4°C, the supernatant was collected into a new tube. The protein contents of all lysates were determined using the Bradford assay (Bio-Rad Laboratories, Hercules, CA, USA). Lysates of 45 μg of total protein were boiled in 2 × sample buffer (125 mM Tris-HCl pH 6.8, 4% SDS, 20% glycerol, 100 mM DTT, 1% bromophenol blue) for 5 min. The proteins were resolved on SDS-PAGE and then electrophoretically transferred to polyvinyl fluoride membranes. After blocking with Blocking One or Blocking One-P (Nacalai Tesque, Kyoto, Japan) for 30 min, the membranes were incubated with primary antibodies at 4°C overnight, washed with TBS-T (1×TBS with 0.1% Tween-20) and incubated with secondary antibodies at room temperature for 2 hours. The following primary antibodies were used: α-tubulin (Sigma, #T6074), 4E-BP1 (Cell Signaling, #9452), P-4E-BP1-Thr37/46 (Cell Signaling, #2855), p70S6K (BD Biosciences, #611260), P-p70S6K-Thr389 (Cell Signaling, #9206), mTOR (Cell Signaling, #2972), Raptor (Cell Signaling, #2280), GβL (Cell Signaling, #3274), PI3K (BD Biosciences, #610045), and Akt (Cell Signaling, #9272). The following secondary antibodies were used: horseradish peroxidase-conjugated anti-rabbit IgG antibody, and horseradish peroxidase-conjugated anti-mouse IgG antibody (Cell Signaling, #NA934 and #NA931, respectively). Specific band intensities were detected using ECL Prime chemiluminescence western blotting reagents (GE-Healthcare, Buckingham, UK), quantified with a LAS-3000 mini in combination with Multi Gauge software (version 3.0; Fujifilm) and are shown as arbitrary units (AUs) that were normalized to the intensity of α-tubulin.

### Quantitative RT-PCR

Total RNAs were extracted from the soleus and EDL muscles using RNeasy Fibrous Tissue Mini Kits (Qiagen, Tokyo, Japan) according to the manufacturer’s instructions. Reverse transcription reactions were performed at 42°C for 60 min with 2 μg of the total RNA in a 25-μl reaction volume. The cDNA obtained by the reverse transcription was diluted 1:30, and 3 μl of the diluted solution was used as a template in the real-time quantitative PCR (RT-PCR). RT-PCR analysis with the Thunderbird SYBR PCR mix (Toyobo, Osaka, Japan) was performed with a MyiQ RT-PCR Detection System (Bio-Rad, Hercules, CA). RT-PCR was performed in the following cycles: 60 s at 95°C, followed by 40 cycles of 15 s at 95°C, 15 s at 60°C, and 45 s at 72°C. All data were normalized to β-actin, and quantitative determinations were obtained by the ΔΔCt method. The primer sequences used were as follows: β-actin (forward primer: 5'-TGATGTATGAAGGCTTTGG-3’, reverse primer: 5'-GTT TGTGTAAGGTAAGGTGTG-3’); glucocorticoid receptor (GR) (forward primer: 5'- TACCACAGCTCACCCCTACC-3’, reverse primer: 5'- AGCAGGGTCATTTGGTCATC-3’); Bnip3 (forward primer: 5'-CAGAGCGGGGAGGAGAAC-3’, reverse primer: 5'-GAAGCTGGAACGCTGCTC-3’); MuRF1 (forward primer: 5'-ACATCTTCCAGGCTGCCAAT-3’, reverse primer: 5'-GTTCTCCACCAGCAGGTTCC-3’); atrogin-1 (forward primer: 5'-GAACATCATGCAGAGGCTGA-3’, reverse primer: 5'-GTAGCCGGTCTTCACTGAGC-3’); REDD1 (forward primer: 5'- TAGTGCCCACCTTTCAGTTG-3’, reverse primer: 5'-GTCAGGGACTGGCTGTAACC-3’); REDD2 (forward primer: 5'-GAAACAGAGCCGTTGACCAT-3’, reverse primer: 5'-TTCAAACACCACCTCGTTGA-3’); FoxO1 (forward primer: 5'-CACACA GCTGGGTGTCAGGCTA-3’, reverse primer: 5'-GGGGTGAAGGGCATCTTT-3’); FoxO3 (forward primer: 5'-TTCAAGGATAAGGGCGACAG-3’, reverse primer: 5'-GGCTGTGCAGTGACAGGTT-3’); FoxO4 (forward primer: 5'-GACAAGGGTGACAGCAACAG-3’, reverse primer: 5'-TTGCTGTGCAAAGACAGGTT-3’); Myostatin (forward primer: 5'-CCTGGAAACAGCGCCTAACA-3’, reverse primer: 5'-CGTCACTGCTGTCATCCCTC-3’) and IGF-I (forward primer: 5'-CTTGAGCAACCTGCAAAACA-3’, reverse primer: 5'-GGAAATGCCCATCTCTGAAA-3’).

### Statistical analyses

The data are expressed as the means ± the SEMs. Differences were determined with two-way ANOVAs followed by Tukey-Kramer tests. For the comparison of two values (the comparison of GR mRNA level between soleus and EDL muscles in SDRs, the comparison of ubiquitin-proteasome-related and Dex-induced mRNA levels between SDR and GH-treated SDR muscles and the comparison of contents of mTORC1 components and upstream and downstream molecules of mTORC1 between SDR and SD rat muscles), student’s t test was used. P < 0.05 was considered to be significant.

## Results

### Effects of BCAA and/or Dex administration on body weight, food intake and muscle mass in the SDRs

**Fig 1:**
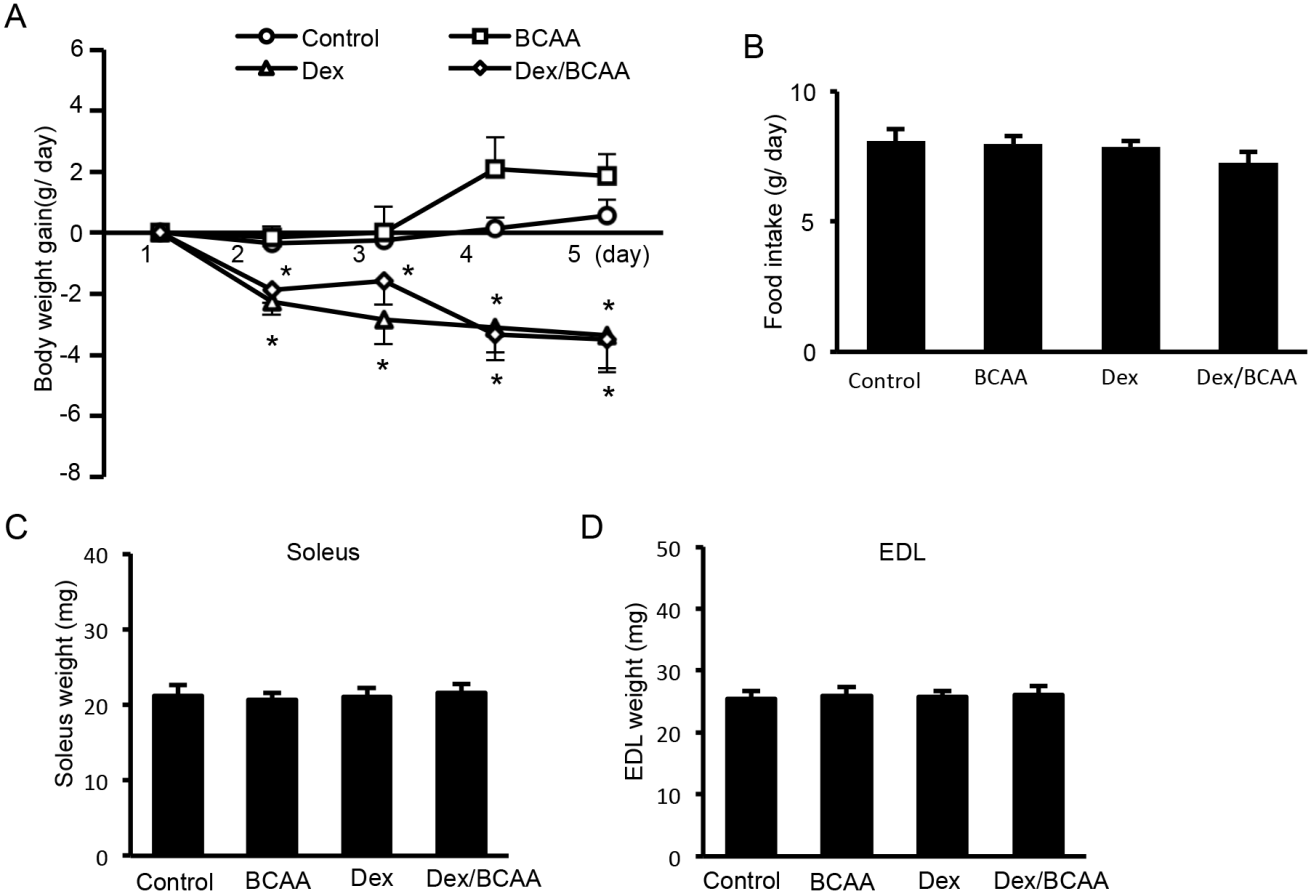
Effect of BCAA and Dex administration on body weight, food intake and muscle mass in the SDRs. A. Treatment with Dex for 5 days elicited a reduction in body weight gain in the SDRs irrespective of BCAA administration. BCAA did not increase body weight gain. B, C, D. Neither Dex nor BCAA affected food intake, soleus or EDL muscle mass. *, *P* < 0.05 vs. control group.

Dex treatment for 5 days produced a reduction in body weight gain in the SDRs regardless of BCAA administration compared to the controls. BCAA did not increase body weight gain (Fig 1A). However, neither Dex nor BCAA affected food intake or soleus or EDL muscle mass (Fig 1B, 1C and 1D).

### Effects of BCAA and/or Dex administration on skeletal muscle fiber size in the SDRs

**Fig 2:**
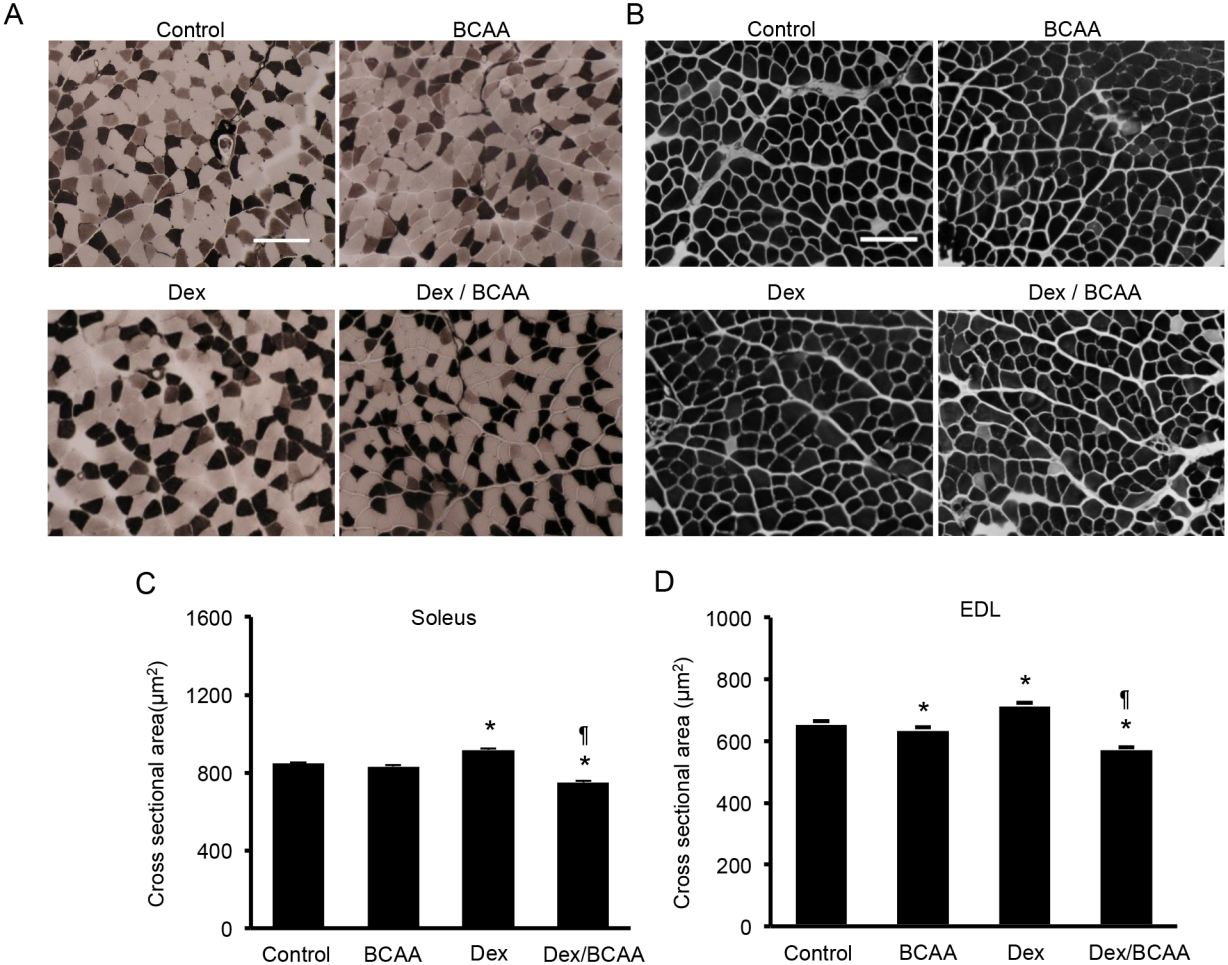
The CSAs of the muscle fibers in the soleus and EDL muscles of the SDRs treated with dexamethasone, BCAA or both. A. ATPase staining (pH 10.7) of soleus muscle fibers from the SDRs. In this staining, type 1 fibers are stained light, and type 2 fibers are dark. Scale bar: 100 μm. B. ATPase staining (pH 10.7) of EDL muscle fibers from the SDRs. C. BCAA did not increase the CSAs in the soleus muscles. Dex elicited a slight increment in the CSAs compared to the control. The administration of both Dex and BCAA resulted in a decrease in CSA compared to the Dex-treated SDRs. *, *P* < 0.05 vs. control group; ¶, *P* < 0.05 vs. Dex-treated group. D. BCAA did not increase but rather decreased the CSAs in the EDL muscles. Dex elicited a slight increment in CSA, and the administration of both Dex and BCAA resulted in a decrease in CSA compared to the control and Dex-treated SDRs, respectively. *, *P* < 0.05 vs. control group; ¶, *P* < 0.05 vs. Dex-treated group.

We measured the CSAs of muscle fibers from the soleus and EDL muscles of BCAA- and/or Dex-treated SDRs. In the soleus muscles, BCAA did not alter the CSAs of the muscle fibers in the absence of Dex. Dex treatment produced a slight increment in muscle fiber CSA, and BCAA decreased muscle fiber CSA in the presence of Dex (control; 848 ± 6.5 μm^2^, BCAA; 832 ± 6.6 μm^2^, Dex; 917 ± 6.3 μm^2^, Dex + BCAA; 751 ± 6.1 μm^2^, Fig 2A and 2C). In the EDL muscles, Dex increased the muscle fiber CSA. BCAA decreased the CSA in the presence and absence of Dex (control; 659 ± 4.7 μm^2^, BCAA; 640 ± 4.7 μm^2^, Dex; 719 ± 4.8 μm^2^, Dex + BCAA; 576 ± 3.7 μm^2^, Fig 2B and 2D). Type 1 fibers and type 2 fibers in soleus and EDL muscles showed similar responses to BCAA and Dex (S1 Table).

### Effects of BCAA administration on the phosphorylations of p70S6K and 4E-BP1 in the SDR skeletal muscles

**Fig 3:**
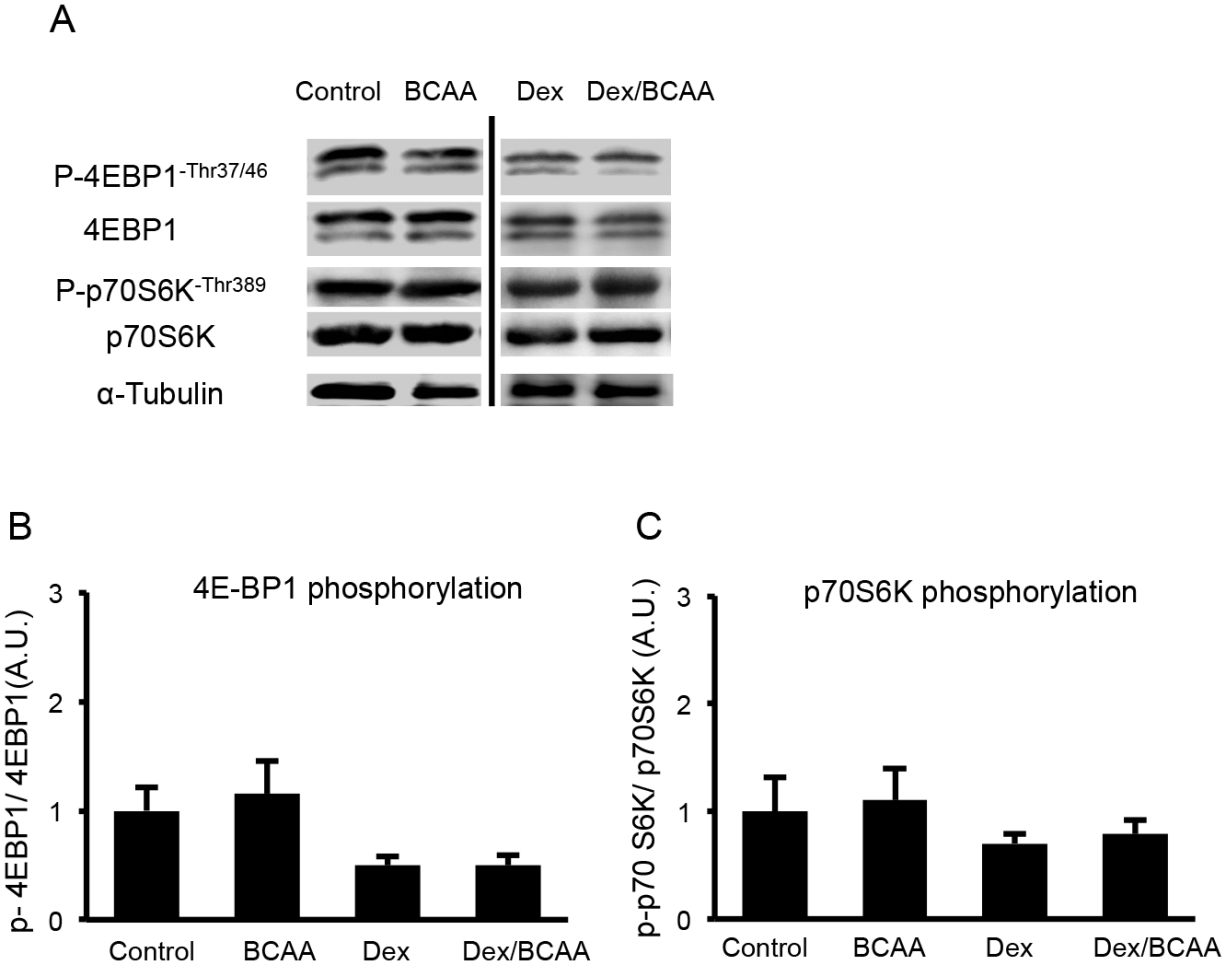
Phosphorylations of 4E-BP1 and p70S6K in soleus muscles of the SDR. A. Western blots for phospho (Thr37/46) and total 4E-BP1 and phospho (Thr389) and total p70S6K. B. BCAA did not increase the phosphorylation of 4E-BP1 in the SDR muscles in the presence or absence of Dex. C. BCAA did not increase the phosphorylation of p70S6K in the presence or absence of Dex.

Based on the results concerning the CSAs of the muscle fibers of the SDRs, BCAA appeared to lose its hypertrophic effect on muscles that had been reported in SD rats. Therefore, we examined whether BCAA stimulated the phosphorylations of p70S6K and 4E-BP1, which are downstream molecules of mTOR. In this experiment, BCAA administration did not increase the phosphorylation of either p70S6K (Thr389) or 4E-BP1 (Thr37/46) compared to the controls in the absence and presence of Dex. Dex showed a tendency to decrease the phosphorylation of p70S6K and 4E-BP1, but it was not significant (Fig 3).

### Effects of BCAA and/or Dex administration on autophagy-related, ubiquitin-proteasome-related and Dex-induced gene expressions in the SDR skeletal muscles

**Fig 4:**
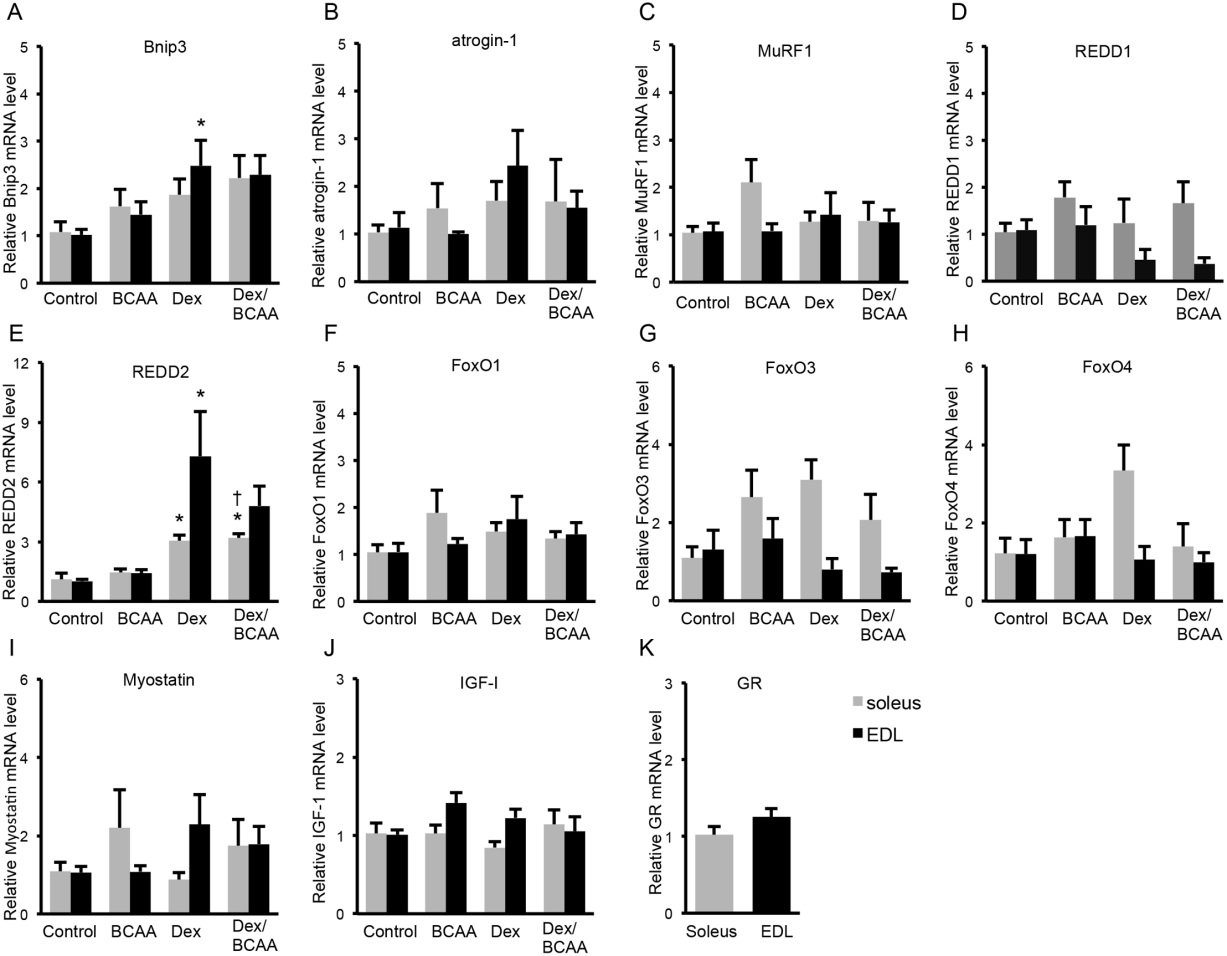
Effects of BCAA on the dexamethasone-induced protein degradation pathway and GR mRNA levels in the SDR muscles. A. Dex increased Bnip3 mRNA level, but BCAA did not influence the Dex-elevated Bnip3 mRNA level in the EDL muscles of SDRs. Dex caused a similar response in the soleus muscles, but it was not significant. B, C and D. Neither Dex nor BCAA exhibited a significant influence on atrogin-1, MuRF1 or REDD1 mRNA levels. E. Dex increased the REDD2 mRNA level. BCAA exhibited a tendency to suppress the overexpression of REDD2 induced by Dex, but this difference was not significant. F, G, H, I and J. Neither Dex nor BCAA exhibited a significant influence on FoxO1, FoxO3, FoxO4, myostatin or IGF-I mRNA levels. K. GR mRNA level was not different between soleus and EDL muscles. Black column: EDL muscles, gray column: soleus muscles. *, *P* < 0.05 vs. control group; +, *P* < 0.05 vs. BCAA-treated group.

We examined Bnip3 mRNA levels as a pro-apoptotic protein that can induce autophagy. Dex increased the Bnip3 mRNA level, but BCAA did not influence the Dex-induced elevation of Bnip3 mRNA in the EDL muscles of SDRs. Dex caused a similar response in the soleus muscles, but it was not significant. (Fig 4A). In contrast, neither Dex nor BCAA exhibited a significant influence on atrogin-1 or MuRF1 mRNA levels, which were examined as markers of the activation of the ubiquitin-proteasome pathway; however, Dex tended to increase atrogin-1 mRNA (Fig 4B and 4C). REDD1 and REED2 are inhibitors of mTOR signaling, and REDD1 is reportedly induced by Dex. In the present study, Dex increased REDD2 mRNA but not REDD1 mRNA (Fig 4D and 4E). BCAA tended to reduce the Dex-induced elevation of REDD2 mRNA, although this effect was not significant. Neither Dex nor BCAA exhibited a significant influence on FoxO1, FoxO3, FoxO4 or myostatin mRNA levels (Fig 4F, 4G, 4H and 4I), although these mRNAs have been reported to increase in response to Dex. Dex and BCAA did not influence IGF-I mRNA levels (Fig 4J). GR mRNA levels were not different between soleus and EDL muscles (Fig 4K).

### Effects of BCAA and/or Dex on body weight, food intake and muscle mass in the GH-treated SDRs

**Fig 5:**
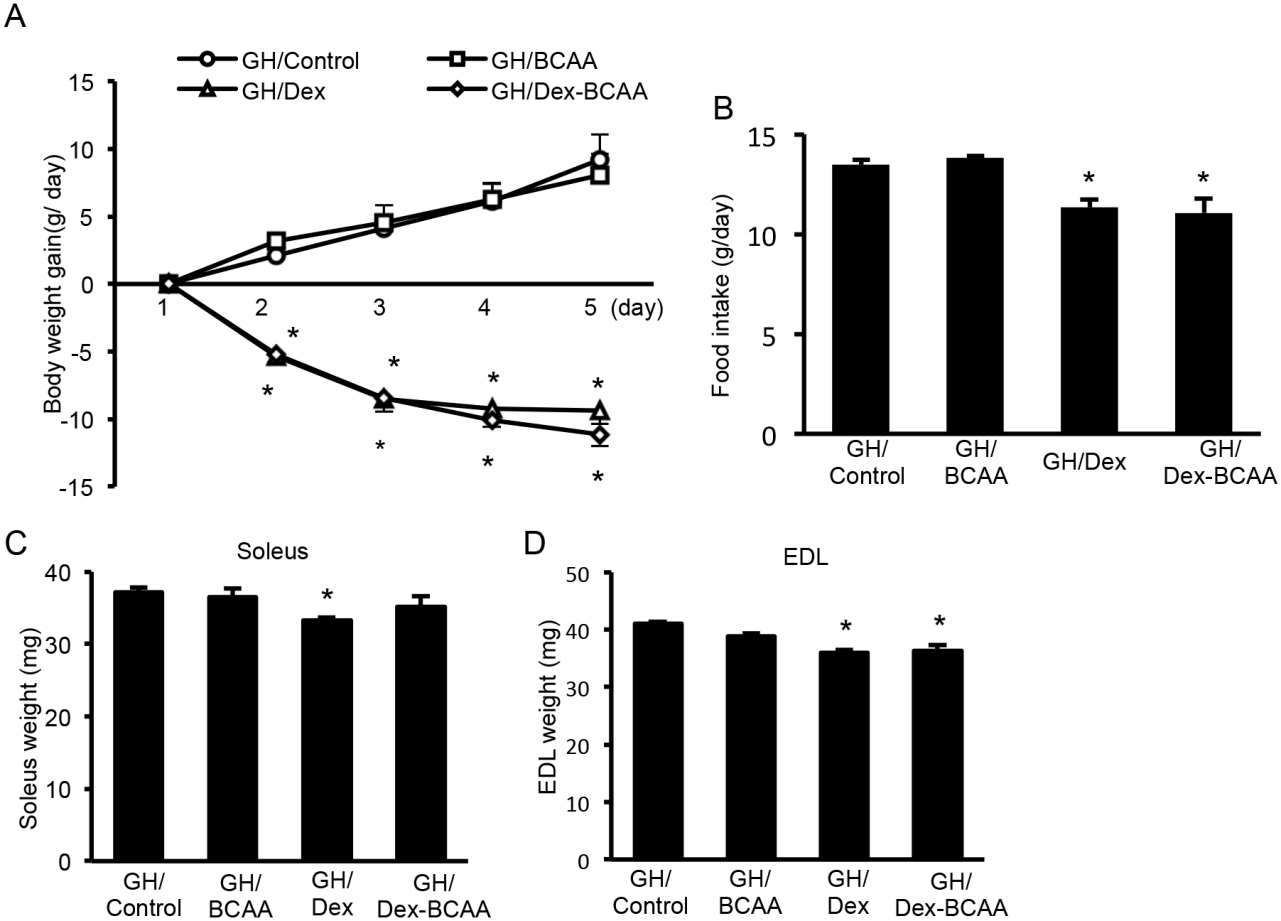
Effect of BCAA and Dex administration on body weight, food intake and muscle mass in the GH-treated SDRs. A. Dex treatment for 5 days elicited a reduction in body weight gain in the GH-administered SDRs irrespective of BCAA administration. BCAA did not increase body weight gain. B. Food intake by the Dex-treated SDRs was decreased irrespective of BCAA administration. C. Dex reduced soleus muscle mass, and BCAA recovered this reduction. D. Dex reduced EDL muscle mass, but BCAA did not recover this reduction. *, *P* < 0.05 vs. control group.

To examine the effect of GH on SDR muscles, we administered GH to the SDRs using osmotic pumps. GH increased the body weights of SDRs in experiment 2 compared to the SDRs that did not receive GH in experiment 1 (control; 58.1 ± 2.1 g, GH/control; 98.8 ± 2.6 g, at 8 weeks). However, Dex administration for 5 days elicited a reduction in body weight gain in the GH-treated SDRs irrespective of the administration of BCAA (body weight of GH/control; 98.8 ± 2.6 g, GH/Dex; 88.4 ± 0.7 g, GH/Dex + BCAA; 87.8 ± 1.0 g at 8 weeks). BCAA did not increase body weight gain (Fig 5A). Dex decreased food intake irrespective of BCAA administration (Fig 5B). Although Dex decreased the soleus muscle weights, BCAA reversed this decrease (Fig 5C). Dex decreased the EDL muscle weights in the presence and absence of BCAA (Fig 5D).

### Effects of BCAA and/or Dex on the CSAs of the muscle fibers in the GH-treated SDRs

**Fig 6:**
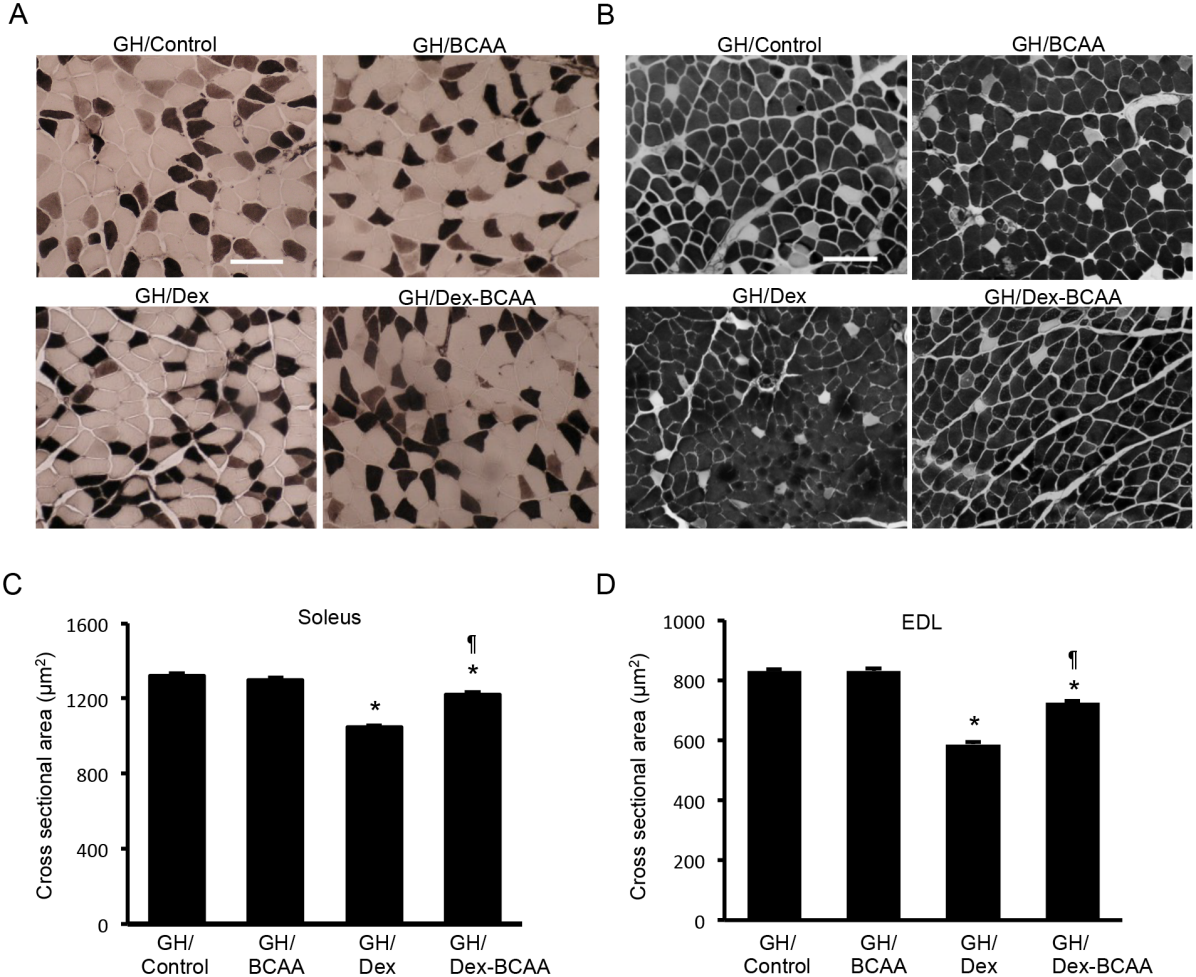
Muscle fiber CSAs of the soleus and EDL muscles in the GH-administered SDRs treated with dexamethasone, BCAA or both. A. ATPase staining (pH 10.7) of the soleus muscle fibers in GH-treated SDRs. In this staining, type 1 fiber are stained light, and type 2 fibers are dark. Scale bar: 100 μm. B. ATPase staining (pH 10.7) of the EDL muscle fibers of the GH-treated SDRs. C. Dex elicited a decrease in the CSA of the soleus muscles, and BCAA significantly recovered this decrease in CSA in the Dex-treated SDRs when supplemented with GH. *, *P* < 0.05 vs. control group; ¶, *P* < 0.05 vs. Dex-treated group. D. Dex elicited a decrease in the CSAs of the EDL muscles in the GH-treated SDRs. BCAA partially but significantly recovered the decrease in CSA.

In contrast to the SDRs without GH treatment, in the GH-treated SDRs, Dex markedly decreased the CSAs of the muscle fibers of the soleus (GH/control; 1319 ± 16.9 μm^2^, GH/Dex; 1046 ± 8.5 μm^2^) and EDL muscles (GH/control; 829 ± 6.4 μm^2^, GH/Dex; 587 ± 8.5 μm^2^). BCAA significantly reduced the Dex-induced decrease in CSA in the soleus (GH/Dex; 1046 ± 8.5 μm^2^, GH/Dex + BCAA; 1222 ± 12.3 μm^2^) and EDL muscles (GH/Dex; 587 ± 8.5 μm^2^, GH/Dex + BCAA; 725 ± 6.4 μm^2^) in the GH-treated SDRs, but BCAA alone did not increase the CSA in the soleus (GH/control; 1319 ± 16.9 μm^2^, GH/BCAA; 1298 ± 14.4 μm^2^) or EDL muscle (GH/control; 829 ± 6.4 μm^2^, GH/BCAA; 831 ± 8.6 μm^2^) compared to the control (Fig 6). The effect of Dex on muscle atrophy was similarly potent in soleus and EDL muscles and independent on muscle fiber type. Type 1 fibers and type 2 fibers in soleus and EDL muscles showed similar responses to BCAA and Dex (S1 Table).

### Effects of BCAA administration on the phosphorylations of p70S6K and 4E-BP1 in the GH-treated SDR skeletal muscles

**Fig 7:**
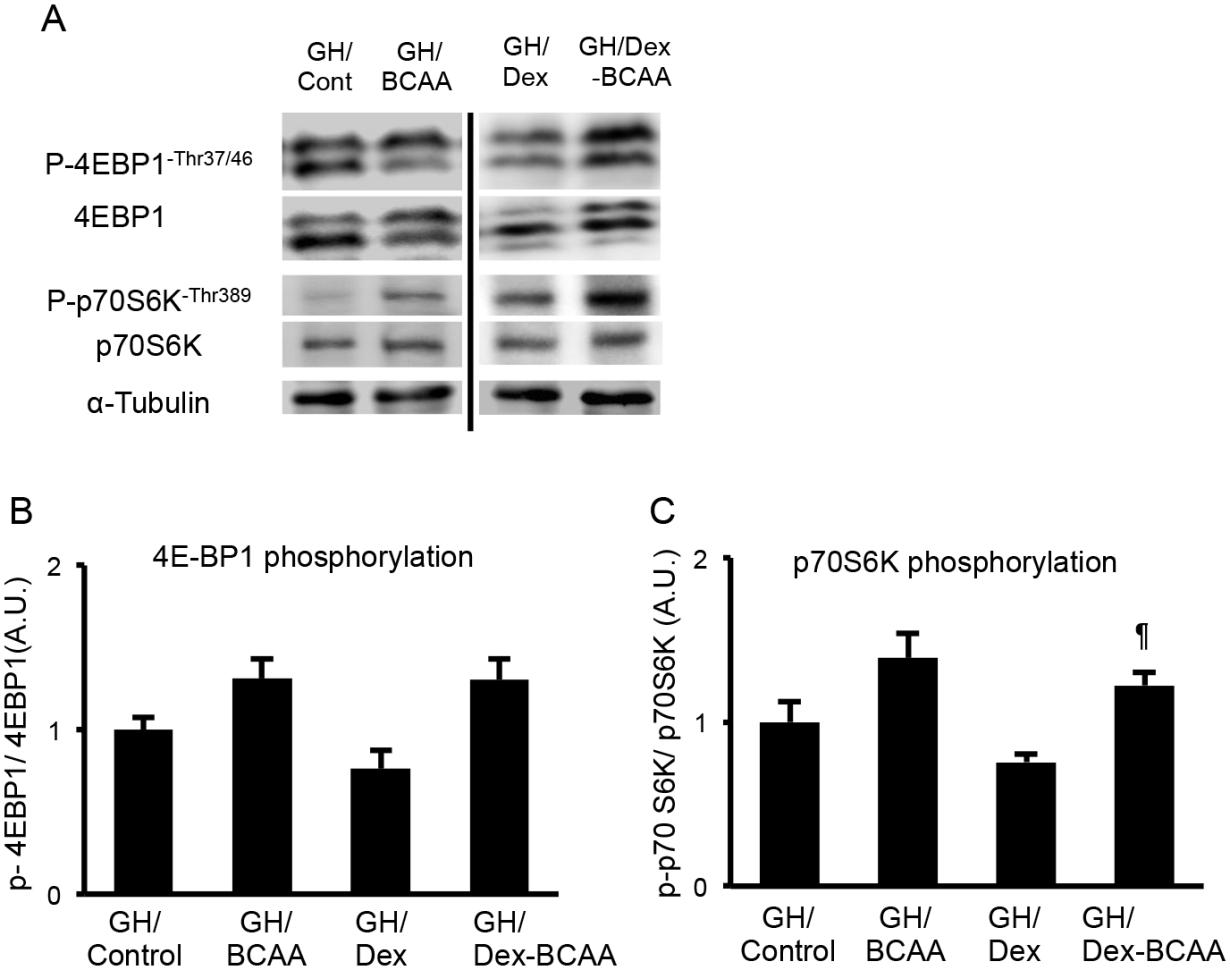
Phosphorylations of 4E-BP1 and p70S6K in the soleus muscles of the GH-administered SDRs. A. Western blots for phospho (Thr37/46) and total 4E-BP1 and phospho (Thr389) and total p70S6K in the GH-administered SDRs. B. BCAA tended to increase the phosphorylation of 4E-BP1 in the GH-treated SDR muscles in the presence and absence of Dex. C. BCAA significantly increased the phosphorylation of p70S6K in the presence of Dex and showed a tendency to elevate the phosphorylation in the absence of Dex. ¶, *P* < 0.05 vs. Dex-treated group.

In accordance with the effects of BCAA on Dex-induced muscle atrophy in the GH-treated SDRs, BCAA administration significantly increased the phosphorylation of p70S6K in the GH-treated SDR skeletal muscles in the presence of Dex. BCAA administration showed a tendency to increase the phosphorylation of p70S6K in the absence of Dex and the phosphorylation of 4E-BP1 in the absence and presence of Dex, but these were not significant (Fig 7).

### Effects of BCAA and/or Dex administration on the autophagy-related, ubiquitin-proteasome-related and Dex-induced gene expressions in the GH-treated SDR skeletal muscles

**Fig 8:**
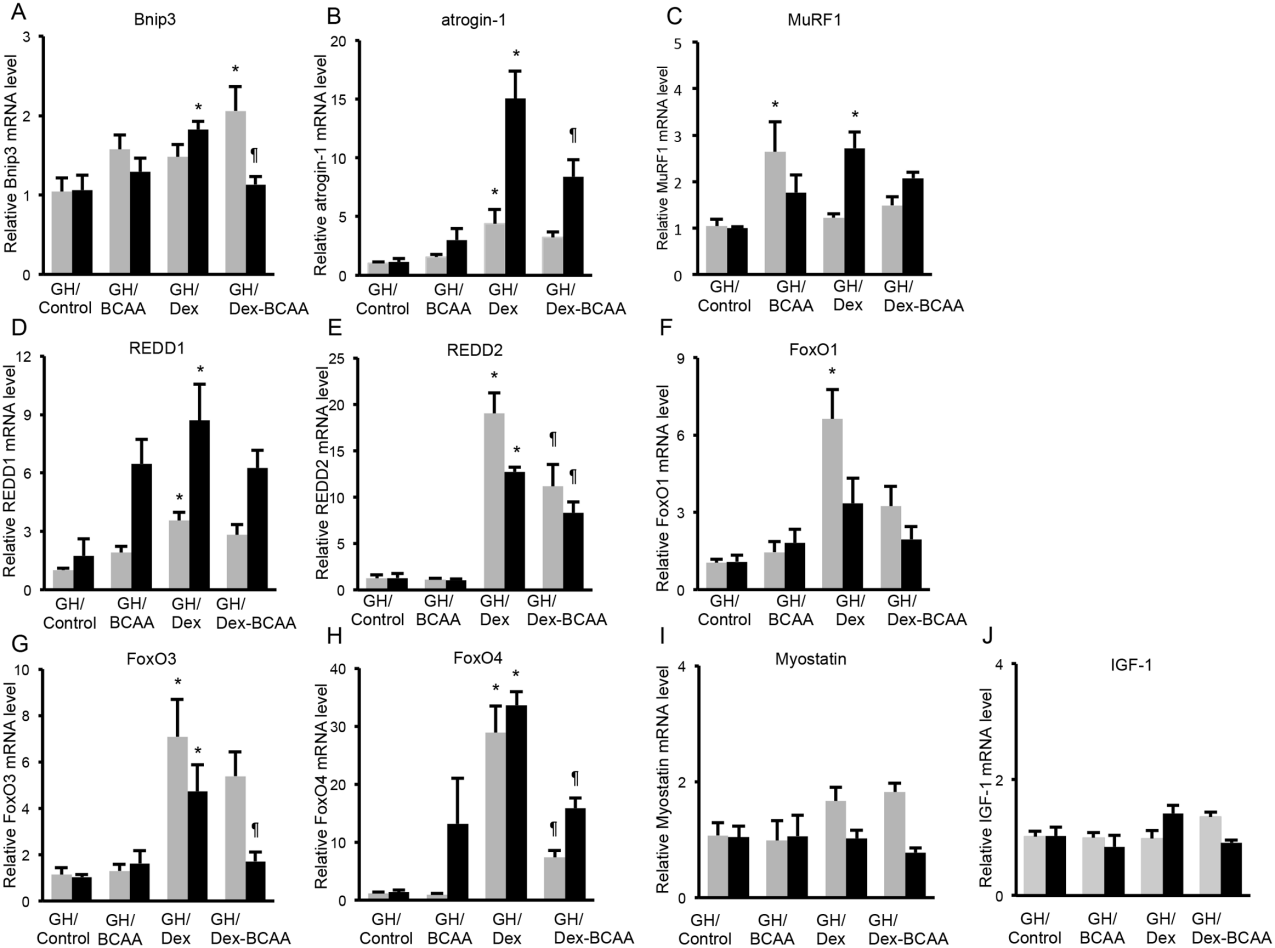
Effect of BCAA on the dexamethasone-induced protein degradation pathway in the GH-treated SDRs. A. In the GH-treated SDR, Dex increased Bnip3 mRNA in EDL muscles, and BCAA reversed this Dex-induced increase in Bnip3 mRNA. BCAA did not influence the Bnip3 mRNA level. B. Dex increased the atrogin-1 mRNA level, and BCAA decreased this Dex-induced increase in atrogin-1 mRNA in EDL muscles. C. Dex increased MuRF1 mRNA in EDL muscles. BCAA exhibited a trend toward decreasing this Dex-induced rise in MuRF1 mRNA, but it was not significant. BCAA increased MuRF1 mRNA in soleus muscles. D and E. Dex increased the REDD1 and REDD2 mRNA levels, and BCAA attenuated the Dex-induced increase in REDD2 mRNA. F, G and H. Dex elevated FoxO3 and FoxO4 mRNA levels and exhibited a tendency to increase the FoxO1 mRNA level. BCAA reversed the Dex-induced elevations of the FoxO3 and FoxO4 mRNA levels. I and J. Neither Dex nor BCAA exhibited a significant influence on myostatin or IGF-I mRNA levels. Black column: EDL muscles, gray column: soleus muscles. *, *P* < 0.05 vs. control group; ¶, *P* < 0.05 vs. Dex-treated group.

Dex increased Bnip3 mRNA, and BCAA significantly suppressed the Dex-induced elevation of Bnip3 mRNA in the EDL muscles of GH-treated SDR. In the soleus muscles, co-administration of Dex and BCAA increased Bnip3 mRNA (Fig 8A). Furthermore, Dex markedly elevated atrogin-1 mRNA, and this increase was significantly decreased by BCAA in the EDL muscles (Fig 8B). Dex increased MuRF1 mRNA levels in the EDL muscles. BCAA exhibited a trend toward reducing the Dex-induced increase in MuRF1 mRNA, although this reduction was not significant (Fig 8C). Dex up-regulated REDD1, REDD2, FoxO3 and FoxO4 mRNAs and tended to increase FoxO1 mRNA in the EDL muscles. REDD2, FoxO3 and FoxO4 mRNAs were suppressed by BCAA (Fig 8D, 8E, 8F, 8G and 8H). Neither Dex nor BCAA exhibited a significant influence on myostatin and IGF-I mRNA levels (Fig 8I and 8J). The response patterns of these mRNA to Dex and/or BCAA in the soleus muscles were similar to those in the EDL muscles except MuRF1. Collectively, GH treatment restored the BCAA signaling pathway in the SDR muscles.

### Effects of GH administration on the ubiquitin-proteasome-related and Dex-induced mRNAs and GR mRNA in the SDR skeletal muscles

**Fig 9:**
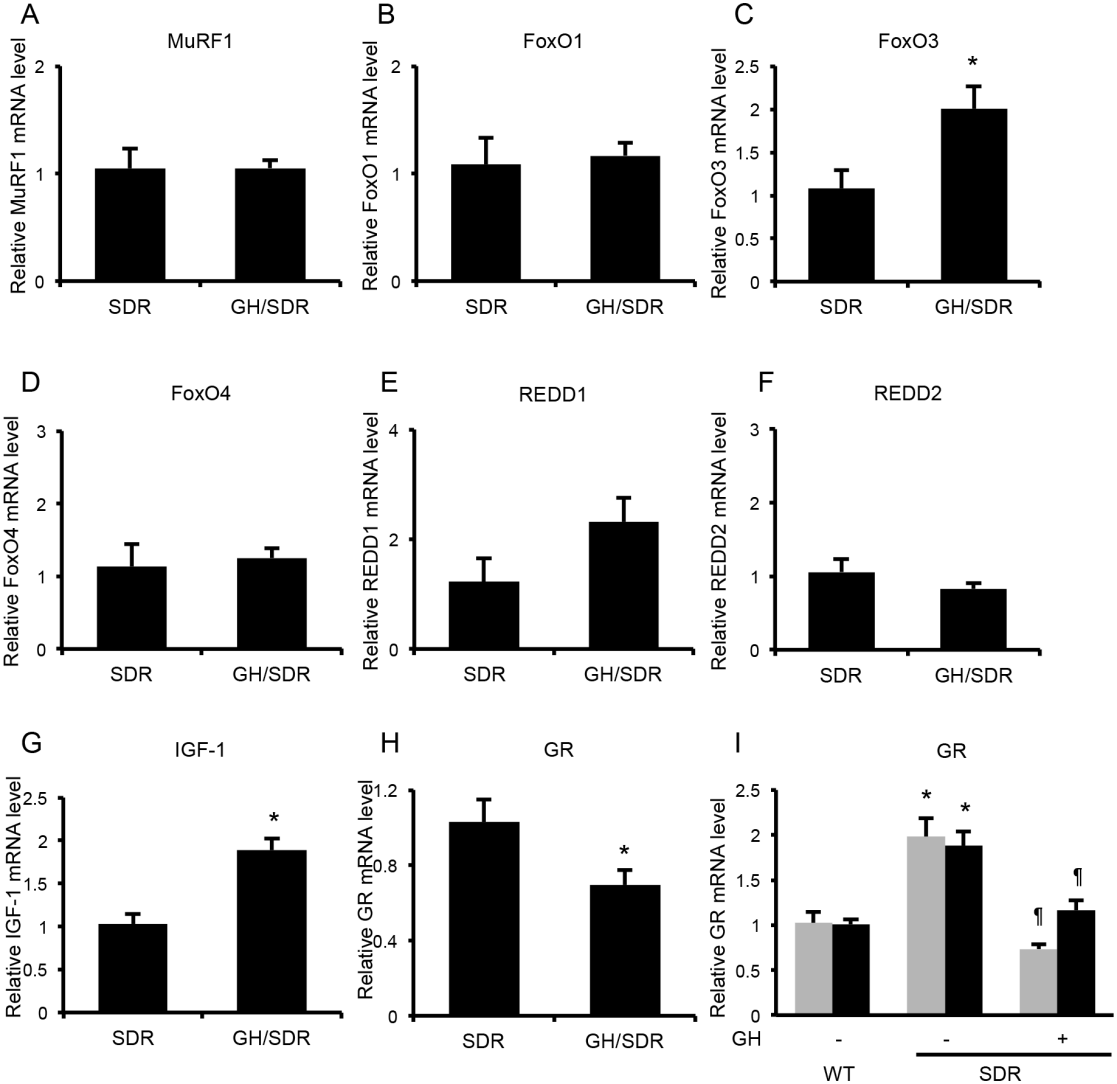
Comparison of basal mRNA levels between the SDR and the GH-treated SDR muscles. A, B, D and F. MuRF1, FoxO1, FoxO4 and REDD2 mRNA levels in rectus femoris muscle were not different between the GH-treated SDRs and the control SDRs. C, E and G. FoxO3, REDD1 and IGF-I mRNA levels were higher in the GH-treated SDRs than in the control SDRs. H. GR mRNA level was lower in the GH-treated SDRs than in the control SDRs. *, *P* < 0.05 vs. control SDRs (A ~ H). I. In soleus (gray column) and EDL muscles (black column), GR mRNA levels were lower in GH-treated SDRs and SD rats than in SDRs. *, *P* < 0.05 vs. normal SD rats (wild); ¶, *P* < 0.05 vs. SDRs.

Effects of Dex and BCAA on ubiquitin-proteasome-related and Dex-induced mRNAs mRNA levels were evident in GH-treated SDR muscles. To examine whether GH affects basal mRNA levels of these, the rectus femoris muscles of the GH-treated SDRs (Experiment 2) and the control SDRs (Experiment 3) were used because of the limited amount of soleus and EDL muscles and basal mRNA levels were compared between the SDRs and the GH-treated SDRs. IGF-I, FoxO3 and REDD1 mRNA levels in rectus femoris muscle were higher in the GH-treated SDRs than in the control SDRs. MuRF1, FoxO1, FoxO4 and REDD2 mRNA levels were not different between the GH-treated SDRs and the control SDRs (Fig 9A–9G).

In the GH-treated SDR muscles, the effect of Dex that stimulates muscle atrophy was restored. To exclude the possibility that GR mRNA expression might have increased in GH-treated SDR muscles, we examined GR mRNA levels. GR mRNA levels were higher in SDRs than in SD rats in rectus femoris muscle. In both soleus and EDL muscles, GR mRNA levels were higher in SDRs than in SD rats. GH-treatment reduced GR mRNA levels in SDR muscles. The GR mRNA levels in GH-treated SDR muscles were not different from those in SD rat muscles (Fig 9H and 9I). This finding suggests that low expression of GR is not the reason of Dex unresponsiveness in SDR muscles.

### Contents of the mTORC1 components and upstream and downstream molecules of mTORC1 in the muscles of the SDR and SD rats

**Fig 10:**
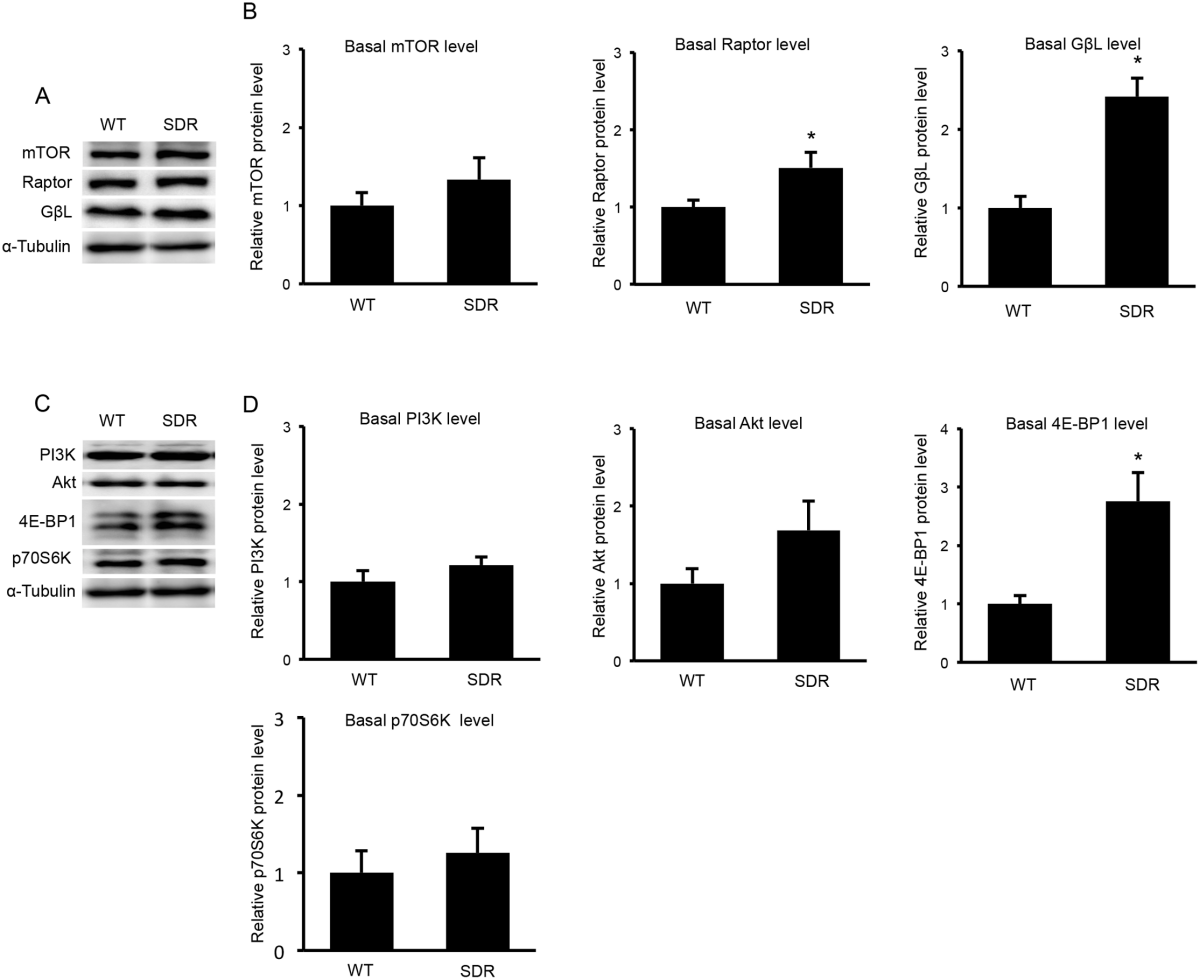
Contents of the mTORC1 components and the signaling molecules up- and downstream of mTORC1 in the soleus muscles of the SDR and SD rats. A. Western blots for mTOR, Raptor and GβL in the SDR and wild-type SD rat muscles. B. The level of mTOR protein in the SDR muscles was not different from that in the SD rats. Raptor and GβL were increased in the SDR muscles compared to the SD rat muscles. C. Western blots for PI3K, Akt, 4E-BP1 and p70S6K in the SDR and SD rat muscles. D. The contents of PI3K and Akt, which are molecules that are upstream of mTORC1, were not different between the SDR and SD rats. The p70S6K content in the SDR muscles was not different from that in the SD rat muscles. The 4E-BP1 protein level was greater in the SDRs than in the SD rat.

mTORC1 plays a pivotal role in the transmission of the action of BCAA. To clarify why BCAA action was not transmitted in the SDR muscle, we compared the mTORC1 components of mTOR, Raptor and GβL between the muscles of the SDRs and the age-matched SD rats. The mTOR protein content did not differ between the SDR and SD rats. The amounts of Raptor and GβL were greater in the SDRs than in the SD rats (Fig 10A and 10B). The contents of PI3K and Akt, which are molecules upstream of mTORC1 were not different between the SDR and SD rats. The amount of p70S6K, which is downstream of mTORC1, in the SDRs was similar to that in the SD rats. The 4E-BP1 content was higher in the SDR than in the SD rats (Fig 10C and 10D). These results suggest that the altered function of mTORC1 was not due to reductions in the contents of these signaling molecules.

## Discussion

In the present study, we found that Dex failed to modulate muscle mass in SDRs. In the SDRs, Dex did not decrease the CSAs of the muscle fibers but did decrease these CSAs after GH administration. Dex did not elevate atrogin-1 or MuRF1 mRNA levels in the SDR muscles, but Dex did increase the levels of these mRNAs in the muscles of the normal SD rats [7, 24]. However, following GH administration, the increases in atrogin-1 and MuRF1 were observed in the SDRs. These findings are consistent with the responses of the REDD1, FoxO3 and FoxO4 mRNAs. These mRNAs have been reported to be increased by Dex in normal rats [7, 8]. In the present study, Dex did not increase REDD1, FoxO3 or FoxO4 mRNAs in the SDRs but did increase these mRNAs after GH administration. Britto et al. reported that REDD1 protein expression was increased 5 hours after the Dex administration but not detected 24 hours after the administration, indicating the time-dependent expression of REDD1 [31]. Because the muscles were removed 18 hours after the last Dex administration in the present study, REDD1 expressions might have been reduced at the sampling of SDR muscles. However, in contrast to SDR muscles in which REDD1 mRNA was not increased by Dex, REDD1 mRNA level was markedly induced by Dex in GH-treated SDR muscles. This result indicates that Dex response was restored after GH-treatment.

There are some discrepancies in REDD2 and myostatin mRNA expressions between previous reports and our results. In the present study, we found that Dex stimulated REDD2 expression. Little is known about the effect of Dex on REDD2 mRNA levels. To the best of our knowledge, effect of Dex on REDD2 mRNA expression in muscles was reported in only one article and Dex had no effect on REDD2 mRNA levels in the report [8]. The reason that causes the difference is unknown. It is reported that glucocorticoids increase myostatin mRNA in rat muscles and cultured muscle cells [29]. However, Jesinkey et al. reported that Dex treatment for 7 days did not stimulate myostatin mRNA levels in muscles [32], which is consistent with our result. In the present study, 5 and 6 SDRs per one group were used in the Experiment 1 and 2, respectively. The small number of SDRs due to limited availability of SDRs might explain the difference in myostatin mRNA. Some mRNA levels were not significantly influenced by Dex and BCAA, showing just a tendency. These responses also might be explained by the limited number of SDRs. It is difficult to exclude the possibility of the type II error that underestimates significant results.

We found that GR mRNA level was higher in the SDR muscles than in the GH-administered SDR muscles or in the normal SD rat muscles. This result suggests that the defect of Dex’s action in SDR muscles is not due to the low expression of GR. Furthermore, Dex administration resulted in decreased body weight in SDR irrespective of BCAA or GH administration, indicating bioactivity of Dex was preserved in SDRs. Dex might disturb the pathways that are activated by GH in the muscles, and the actions of Dex on the muscles might have been unobservable in the absence of GH. A previous up-regulation of CSA by GH might be required for the suppressive effect of Dex, and previous down-regulations of atrogin-1, MuRF1, REDD1, FoxO3 and FoxO4 mRNA levels by GH might be required for the increase in these mRNA levels that is mediated by Dex. However, basal MuRF1, REDD1, FoxO3 and FoxO4 mRNA levels were not decreased in the GH-treated SDRs compared to the SDRs, suggesting other mechanism may regulate the Dex unresponsiveness.

Dex induced muscle atrophy in soleus and EDL muscles after GH administration in the present study. The effect was observed in both type 1 and type 2 fibers. There are many reports that Dex stimulated muscles atrophy in EDL not but soleus muscle [7, 33]. In contrast, there are many reports including ours that Dex caused muscle atrophy in both EDL and soleus muscles [24, 34–37]. Consistent with the Dex’s action in EDL and soleus muscles, we found that GR mRNA levels were not different between soleus and EDL muscles.

We also found that the effect of BCAA on muscle mass was not clear in SDRs. BCAA did not increase CSA of muscle fibers in SDR. This finding contrasted our previous finding that BCAA increases CSA of muscle fibers in SD rats [24]. Furthermore, we found that, in the SDR muscles, BCAA did not stimulate the phosphorylation of p70S6K or 4E-BP1, both of which are important for enhancing protein synthesis [23]. Additionally, the protein degradation pathway did not appear to be affected by BCAA in the SDR muscles. It has been reported that Bnip3, atrogin-1 and MuRF1 expressions are increased in a variety of types of muscle atrophy and stimulate muscle atrophy via the activation of the autophagy and ubiquitin-proteasome systems [7, 24, 25]. Many reports including our own indicate that Dex increases the mRNAs of these autophagy- and ubiquitin-proteasome-related proteins [38–40] and that BCAA suppresses these increases [7, 24]. In contrast to these previous studies, BCAA did not affect Bnip3, atrogin-1 or MuRF1 mRNA levels in the present study. Collectively, BCAA appeared to lose its bioactivity in SDR muscles.

GH-treatment restored the actions of BCAA on the SDR muscles. After the GH administration, Dex decreased the CSA of SDR muscle fibers and BCAA suppressed the Dex-induced decrease in CSA in accordance with the recovery of intracellular signal transmission, which included the phosphorylations of p70S6K, suggesting the activation of mTOR. The finding of the present study that mTOR activity was reduced in the SDR muscles compared to the muscles of GH-administered SDR is consistent with those of previous reports. Sharp et al. reported that the phosphorylation of p70S6K is decreased in Ames dwarf mice with hypopituitarism due to Prop1 mutation [41]. mTOR activity has been reported to be lower in other GH-deficient or GH receptor knockout mice than in normal mice [42]. Also, from a point of view of Dex-induced mRNA expressions, mTOR activity appeared to increase in SDR muscles after the GH administration. BCAA has been reported to stimulate mTOR activity to attenuate the Dex-induced increases in Bnip3, atrogin-1 and MuRF1 mRNAs [7]. In the present study, BCAA reduced Bnip3 and atrogin-1 mRNAs in SDR muscles after GH administration, not without GH administration. These findings suggest that mTOR might be a target of GH and that SDRs might exhibit some abnormalities in mTOR function.

To exclude the possibility that the mTORC1 dysfunction was due to the reduced amount of mTOR, we measured mTOR protein levels in SDR and SD rats; however, we found that the mTOR contents were not different between the SDR and SD rat muscles. Moreover, the amounts of Raptor and GβL, both of which are components of mTORC1, were increased in the SDRs compared to SD rats in the present study. These results suggested that the reduced mTORC1 activity was not due to reductions in the quantities of these subunits. The protein levels of PI3K and Akt, which are upstream of mTORC1, were not different between the SD and SDR muscles. The levels of p70S6K and 4E-BP1, which are downstream of mTORC1, in the SDR muscles were not reduced compared to the SD rat muscles. These results indicated that GH affected the function of mTORC1 but not the amounts of mTORC1 or the molecules upstream and downstream of mTORC1. In contrast to our hypothesis, BCAA did not exert a marked effect on muscle mass in GH-deficient rats, and GH was required for the BCAA’s action on muscle mass. Sancak et al. recently reported that amino acids stimulated the interaction of Rag proteins with mTORC1 and that this was necessary for the activation of the mTORC1 pathway by amino acids. The Rag proteins do not directly stimulate the kinase activity of mTORC1, but promote the intracellular localization of mTOR to a compartment that contains its activator Rheb that is a downstream molecule of PI3K [43, 44]. This is an interesting model to explain the interaction of growth factors and BCAA to stimulate mTOR activity and this model might explain our present results.

Although GH increased IGF-I mRNA in the present study, it is not clear whether the recovery of BCAA-induced mTOR activation was due to the direct action of GH or an indirect action via IGF-1. While GH has a direct effect on muscle that is mediated independently of IGF-1 [45, 46], IGF-1 also exerts an action on muscles [21, 47]. Kim et al. reported that GH does not increase muscle mass, the CSAs of muscle fibers or BrdU uptake by muscles in mice that over-express dominant negative IGF-1 receptors under the control of the muscle-specific creatinine kinase [48]. This indicates that GH action in muscle development is mediated by IGF-1. Both locally produced and circulating IGF-1 can act on muscles. Currently, IGF-1 that is locally produced in muscles is believed to be more important than circulating IGF-1 for muscle development and the maintenance of muscle mass based on analyses of several mouse models with reduced IGF-1 signaling [20, 21]. Recently, however, circulating IGF-1 has been reported to exert effects on muscles [49]. Taken together, these findings indicate that the possibility that the IGF-1 might restore BCAA action in SDR muscle cannot be excluded, although it is not known whether the locally produced or circulating IGF-1 is important.

Food consumption was increased in the GH-treated SDRs compared to the SD rats. Because increased food consumption has been reported to increase mTOR activity [50] but not mTOR content, we cannot exclude the possibility that the increased food consumption might have restored the BCAA action in the SDR muscles after the GH treatment. In this context, GH might have an indirect action on the muscle that is mediated via increased food consumption.

The muscle fiber compositions of the SDR and normal SD rats were different. Muscle mass and the CSAs of the muscle fibers were increased by GH administration, but the fiber composition of the SDRs was not altered by GH administration. Type 2 fibers were dominant in the SDR muscles (50.6% type 1 fiber, 49.4% type 2 fiber in the soleus and 0.7% type 1 fiber, 99.3% type 2 fiber in the EDL). These findings contrasted with the results from the SD rats [24]. In the SD rats, type 1 fibers were dominant in the soleus muscle. Our data about the fiber compositions of the SDRs are consistent with previous reports that hypophysectomy leads to a reduction in type I fiber [51, 52]. However, GH administration after hypophysectomy was reported to restore the muscle fiber composition in this previous study; however, the fiber composition in the SDR muscle was not altered by GH administration in our study.

In conclusion, we found that Dex and BCAA failed to modulate muscle mass in GH-deficient SDRs. BCAA did not stimulate the phosphorylations of p70S6K and did not affect Bnip3 or atrogin-1 mRNA level in the SDR muscles. These abnormalities in the BCAA signaling pathway were restored in the GH-treated SDR muscles. The effect of Dex was also recovered in GH-treated SDR muscles. Dex stimulated muscle atrophy and the atrophy was reversed by BCAA in accordance with the recovery of the BCAA signal pathway. These results indicate that GH is required for the actions of Dex and BCAA in muscles.

## Supporting Information

S1 Table Effect of BCAA and/or Dex on CSA of type1 and type2 muscle fibers in GH-treated or -not treated SDR muscles. Dex decreased CSA of type1 and type2 muscle fibers in GH-treated SDRs, although Dex did not decrease CSA of type1 and type2 muscle fibers in SDRs. BCAA restored the Dex-induced decrease in CSA of type1 and type2 muscle fibers in GH-treated SDRs.
(PDF)
