# Supplementary material for: Dexamethasone and BCAA Failed to Modulate Muscle Mass and mTOR Signaling in GH-Deficient Rats
Source: PLoS One. 2015 Jun 18;10(6):e0128805. doi: 10.1371/journal.pone.0128805 (PMC4472719; doi:10.1371/journal.pone.0128805)
Supplement: S1 Table — Dex decreased CSA of type1 and type2 muscle fibers in GH-treated SDRs, although Dex did not decrease CSA of type1 and type2 muscle fibers in SDRs. BCAA restored the Dex-induced decrease in CSA of type1 and type2 muscle fibers in GH-treated SDRs. (PDF) [file pone.0128805.s001.pdf]

SDR soleus muscles

| | type 1 fiber (μm2) | type 2 fiber (μm2) |
|---|---|---|
| control | 996 ± 8.7 | 694 ± 5.1 |
| BCAA | 1008 ± 8.3 | 633 ± 5.2 |
| Dex | 1051 ± 8.5 | 759 ± 5.9 |
| BCAA + Dex | 899 ± 4.6 | 752 ± 6.1 |

SDR EDL muscles

| | type 1 fiber (μm2) | type 2 fiber (μm2) |
|---|---|---|
| control | 423 ± 22.4 | 661 ± 4.8 |
| BCAA | 374 ± 18.2 | 642 ± 4.7 |
| Dex | 476 ± 16.3 | 722 ± 4.8 |
| BCAA + Dex | 444 ± 23.0 | 577 ± 3.7 |

GH-treated SDR soleus muscles

| | type 1 fiber (μm2) | type 2 fiber (μm2) |
|---|---|---|
| control | 1569 ± 16.6 | 1067 ± 10.6 |
| BCAA | 1558 ± 11.8 | 1015 ± 8.0 |
| Dex | 1284 ± 10.5 | 775 ± 5.9 |
| BCAA + Dex | 1376 ± 14.6 | 946 ± 9.8 |

GH-treated SDR EDL muscles

| | type 1 fiber (μm2) | type 2 fiber (μm2) |
|---|---|---|
| control | 622 ± 31.2 | 834 ± 8.3 |
| BCAA | 605 ± 26.6 | 837 ± 8.7 |
| Dex | 383 ± 31.3 | 592 ± 8.6 |
| BCAA + Dex | 604 ± 15.0 | 726 ± 6.4 |
